# Lung resection surgery in Jehovah’s Witness patients: a 20-year single-center experience

**DOI:** 10.1186/s13019-022-02024-0

**Published:** 2022-10-20

**Authors:** Andy Chao Hsuan Lee, Mark K. Ferguson, Jessica Scott Donington

**Affiliations:** Section of Thoracic Surgery, The University of Chicago Medicine & Biological Sciences, 5841 S. Maryland Ave, Suite S-546, MC 5047, Chicago, IL 60605 USA

**Keywords:** Lobectomy, Segmentectomy, Wedge resection, Bleeding, Bleeding control, Blood, Lung cancer surgery

## Abstract

**Background:**

The paucity of literature on surgical outcomes of Jehovah’s Witness (JW) patients undergoing lung resection suggests some patients with operable lung cancers may be denied resection. The aim of this study is to better understand perioperative outcomes and long-term cancer survival of JW patients undergoing lung resection.

**Methods:**

All pulmonary resections in JW patients at one institution from 2000 through 2020 were examined. Demographics, comorbidities, operative parameters, and perioperative outcomes were reviewed. Among operations performed for primary non-small cell lung cancer (NSCLC), details regarding staging, extent of resection, additional therapies, recurrence, and survival were abstracted.

**Results:**

Seventeen lung resections were performed in fourteen patients. There were nine anatomic resections and eight wedge resections. Fourteen resections (82%) were approached thoracoscopically, of which 3 of 6 anatomic resections were converted to thoracotomy as compared to 1 of 8 wedge resections. There was one (6%) perioperative death. Ten resections in 8 patients were performed for primary pulmonary malignancies, and two patients underwent procedures for recurrent disease. Median survival for resected NSCLCs (N = 7) was 65 months. Three of 6 patients who survived the immediate perioperative period underwent additional procedures: 2 pulmonary wedge resections for diagnosis and one pleural biopsy.

**Conclusions:**

This series of JW patients undergoing lung resections demonstrates that resections for cancer and inflammatory etiologies can be performed safely in the setting of both primary and re-operative procedures.

## Introduction

Jehovah’s Witnesses (JW) refuse transfusion of blood products, based on Biblical passages citing the admonition to “abstain from food polluted by idols, from sexual immorality, from the meat of strangled animals and from blood” (Acts 15:19–20). This heightens their risk of morbidity and mortality in the event of significant blood loss during operations. Lung resections are major surgical endeavors and carry a small but real risk of important blood loss. Nakamura et al. in 2015 retrospectively analyzed 1336 patients undergoing lung resection, and reported mean estimated intraoperative blood loss of 445 mL in lobectomies and 908 mL in pneumonectomies, noting that blood loss was an independent predictor of overall survival [1].

The major concern regarding JW patients undergoing lung resection is the risk of major blood loss that requires blood transfusion for survival. Igai et al. reported that among patients who underwent thoracoscopic anatomic pulmonary resection at a single center, about 11% of the patients had significant intraoperative vessel injury, of which 55% were in a branch of pulmonary artery [2]. Latif et al. further reported that approximately 10% of patients who underwent pulmonary resection for NSCLC at a single center received perioperative blood transfusion, of which 60% received 2 or more red blood cell units [3]. Given that blood transfusion is standard of care in patients who have substantial perioperative blood loss following lung resection, the additional risk of mortality and morbidity due to major blood loss that JW patients need to accept to undergo lung resection it is currently unknown. The 90-day mortality of lobar resection is as low as 1% [4], yet the risk of massive bleeding requiring blood transfusion for pulmonary resection is about 10%, suggesting that the investigation of surgical outcomes in JW’s undergoing lung resections for NSCLC would help determine the added risk of mortality and morbidity for lung resections without transfusion and help to guide care for this challenging group of patients.

There is a paucity of literature regarding the perioperative and long-term outcomes of JW patients undergoing lung resections. Sporadic case reports exist in the literature and highlight the importance pre-operative coagulopathy work up, intraoperative controlled hypotension, and postoperative close monitoring for signs of active bleeding and limited in vitro testing to prevent blood loss, but provide limited information on peri-operative outcomes [5]. The aim of this study is to examine the perioperative complications and long-term outcomes of JW patients undergoing lung resection for both benign and malignant diseases at a tertiary, academic, JW preferred medical center over a 20-year timespan.

## Method

The study was approved by University of Chicago Institutional Review Board (protocol ID IRB20-1397). Waiver of Consent Process and Consent Documentation was granted. All patients who underwent lung resections and had a religious affiliation of Jehovah’s Witness at our institution over a 20-year period (From January 1, 2000 to October 1, 2020) were identified in our EPIC electronic medical record by using the function “Slicer Dicer”. Demographic information including age, race, gender, and location were recorded. Social, surgical, and medical histories were obtained, including smoking status (current smoker, never smoker, or former smoker, defined as an adult who has smoked at least 100 cigarettes in his or her lifetime but who had quit smoking at the time of operation), previous thoracic surgery, and usage of blood thinners and antiplatelet agents. Intraoperative parameters including estimated blood loss (EBL), extent of lung resection, surgical approach, conversions from minimally invasive to open procedures, and tissue diagnoses were recorded. Perioperative outcomes include hemoglobin (Hgb) drop (based on preoperative Hgb measured within 6 months of operations and postoperative Hgb measured within 1 month of operation), length of hospital stay (LOS), chest tube duration, survival at 6 months as well as the negative outcomes as defined by the Society of Thoracic Surgeons Composite Score: operative mortality (death during the same hospitalization as surgery or within 30 days of the procedure) and presence of at least one of these major complications: pneumonia, acute respiratory distress syndrome, bronchopleural fistula, pulmonary embolus, initial ventilator support greater than 48 h, reintubation/respiratory failure, tracheostomy, myocardial infarction, or unexpected return to the operating room [6].

Details regarding use of neoadjuvant and adjuvant therapies, extent lymph node evaluation, pathological staging, tumor recurrence, and need for re-operation were abstracted for patients undergoing curative-intent resections for NSCLC. These patients were staged using the 8th edition of the TNM staging system. All patient receiving resections for NSCLC with curative intent received postoperative surveillance chest CT’s every 3 months to 6 months in the first two years. 3-year survival was calculated for patients who underwent curative-intent resections for NSCLC.

Continuous variables such as age and smoking pack years are reported as median and interquartile range (IQR). Categorical variables such as surgical approach and diagnosis are reported as number and percentage. Both 6-month survival for all patients and 3-year survival for patients who underwent curative-intent resections for NSCLC were calculated using Kaplan–Meier survival analysis (GraphPad Prism 9.0.2.).

## Results

### Demographics and comorbidities

A total of seventeen lung resections were performed in 14 JW patients during the twenty-year period; three patients underwent two pulmonary resections. Demographics are outlined in Table 1. There were 6 men and 8 women, median age at resection was 58 (IQR 49–67) years. The median travel distance to our center was 18.5 miles (IQR 6.6–31 miles), and the longest travel distance was 56.3 miles. Half of the patients were African American. There were two current smokers (14%) and five former smokers (36%), with median pack years of 8.5 (IQR 4–18.8) among patients who were current or prior smokers. Three patients (21%) were on antiplatelet or anticoagulation therapies at time of operation (Table 1).

**Table 1:** Demographics and comorbidities

	# (%)
Sex	
Male	6 (43)
Female	8 (57)
Race	
African American	7 (50)
Caucasian	4 (29)
Hispanic	1 (7)
Asian	1 (7)
Other	1 (7)
Age in years	
Median (IQR)	58 (49, 67)
Smoking history	
Never	7 (50)
Former	5 (36)
Current	2 (14)
Comorbidities	
Diabetes	4 (29)
Hypertension	9 (64)
COPD	1 (7)
Bronchiectasis	2 (14)
Heart failure	2 (14)
Chronic kidney disease	3 (21)
Cerebrovascular accident	2 (14)
Blood thinners	
Antiplatelet	3 (21)
Anticoagulant	3 (21)

### Perioperative outcomes by types of lung resections

Of the seventeen lung resections, nine (53%) were anatomic (including both lobectomy and segmentectomy) and eight were wedge resections. Seven of the nine anatomic resections were for NSCLC, compared to only three of eight wedge resections. Two wedge resections were in patients who had previous lung resections, both were a contralateral diagnostic wedge resections performed for recurrent NSCLC. Two anatomical resections were performed in a single patient with bronchiectasis and destroyed lung secondary to Mycobacterium avium. Fourteen of the 17 resections (82%) were by VATS. Three of the six (50%) anatomic resections were converted to thoracotomy as compared to only one of the eight (12.5%) wedge resections. The median EBL for the anatomic resection cohort was 125 mL (IQR 75–625 mL), which was higher than median EBL for the wedge resection cohort (7.5 mL; IQR 5–26 mL). The median Hgb decrease was 2.2 (g/dl), median chest tube duration was 2 days and median LOS was 3 days for anatomic resections; the corresponding values for wedge resections were 0.5 (g/dl), 1 day, and 1 day, respectively (Table 2).

**Table 2:** Perioperative outcome: anatomical resection vs non-anatomical resection

	Anatomical resection	Wedge resection
Operations (patients)	9 (8)	8 (8)
Reoperation	1	2
Primary lung cancer	7	3
Benign lung disease	2	2
Metastatic cancer	0	3
Initial approach VATS (%)	6 (66.7%)	8 (100%)
Conversion to thoracotomy (%)	3 (50.0%)	1 (12.5%)
Operative mortality (%)	1 (11.0%)	0 (0%)
Overall survival at 6 months (%)	8/9 (88.8%)	8/8 (100%)
Estimated blood loss median (mL; IQR)	125 (75, 625)	8 (5, 26)
Hemoglobin decrease median (mg/dL; IQR)	2.2 (1.5, 3.5)	0.5 (− 0.1, 1.1)
LOS median (days; IQR)	3 (2, 4)	1 (2, 3)
Chest tube duration median (days; IQR)	2 (2, 3)	1 (2, 3)

*IQR* interquartile; *No.* number; *VATS* video-assisted thoracic surgery

Operative mortality following anatomic resection was one of nine (11%) compared to none of those having wedge resections. One patient in the anatomic resection cohort developed at least one major complication (prolonged initial ventilator support and respiratory failure). There was no major complication in the wedge resection cohort. The only in-hospital mortality in cohort was in a 73-year-old male on chronic anticoagulation with a T3N0 adenocarcinoma and received neoadjuvant chemotherapy. He sustained a pulmonary artery injury during a VATS left upper lobectomy. The procedure was converted to thoracotomy and cell saver was brought to the field, but intraoperative blood loss was 4000 ml. Patient was admitted to ICU post-operatively, and Hgb was 3.8 (g/dl). The patient expired on postoperative day 2.

### Perioperative outcomes by diagnosis

Of the seventeen lung resections, nine were performed in patients with NSCLC and eight were performed for other lung pathologies (Table 3). Eighty-eight percent of NSCLC resections were started by VATS, but half were converted to thoracotomy. In contrast, none of the VATS procedures for other diagnoses was converted to an open operation (Table 3). Reasons for conversion included difficult dissection in two (large tumor size and difficult hilar dissection), inability to identify tumor, and intraoperative hemorrhage (Table 4).

**Table 3:** Perioperative outcome: resection for non-small cell lung cancer vs resection for other pathologies

	NSCLC	Other pathology
Operations (patients)	9 (7)	8 (7)
Reoperation	2	1
Anatomic resection	6	3
Wedge resection	3	5
VATS as initial approach (%)	8 (88%)	6 (75%)
Conversion to thoracotomy (%)	4 (50%)	0 (0%)
Operative mortality (%)	1 (11%)	0 (0%)
Overall survival at 6 months (%)	8/9 (88.8%)	8/8 (100%)
EBL median (IQR)	188 (106, 1188)	40 (8, 88)
Hemoglobin decrease median (gm/dL; IQR)	1.5 (− 0.1, 2.1)	1.1 (0.5, 2.2)
LOS median (days; IQR)	2 (2, 3)	1.5 (1, 3)
Chest tube duration median (days; IQR)	2 (2, 3)	1 (1, 2)

*IQR* interquartile; *NSCLC* non-small cell lung cancer; *VATS* video-assisted thoracic surgery; *EBL* estimated blood loss; *LOS* length of stay

**Table 4:** Operative details for conversions from minimally invasive to open approach

Preoperative diagnosis	Timing of conversion to thoracotomy	Reason for conversion to thoracotomy	Operation performed	Total duration of surgery (min)
Stage IIB LUL adeno	Following PA Injury	For emergent control of pulmonary artery injury	Lobectomy	309
Stage IIA RUL adeno	After video inspection	Due to the size of the tumor, and proximity of the tumor to the fissure	Lobectomy	241
Stage IVA LLL adeno	After video inspection	Inability to identify nodule with minimally invasive approach due to depth in tissue	Wedge Resection	125
Stage IIB LLL adeno	During hilar dissection	Difficult hilar dissection	Lobectomy	135

*Adeno* adenocarcinoma; *LUL* left upper lobe; *RUL* right upper lobe; *LLL* left lower lobe

### Oncologic outcomes in patients with NSCLC

**Fig. 1:**
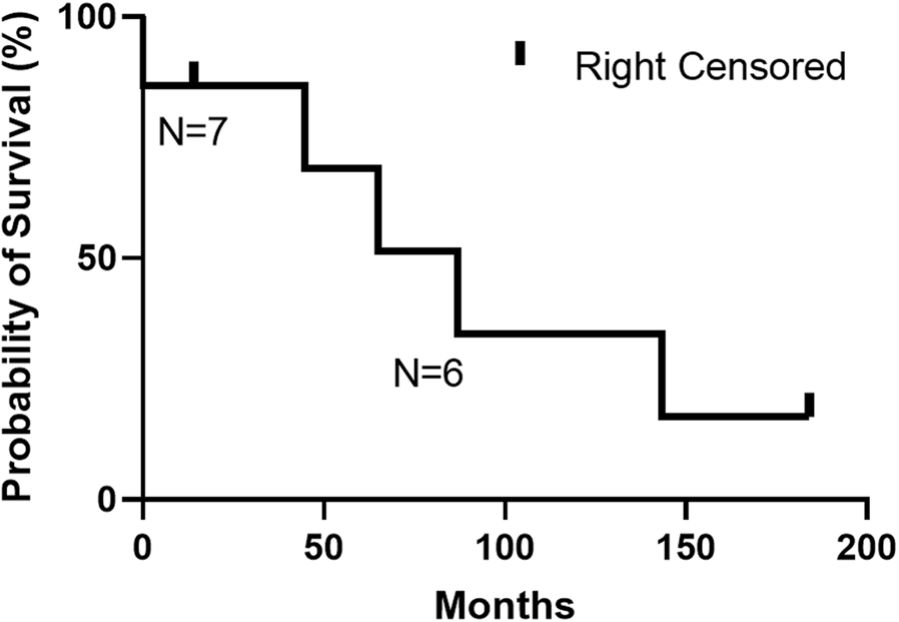
Kaplan Meyer curve for JW patients undergoing curative resections for NSCLC

Seven JW patients underwent curative resections for NSCLC, six for adenocarcinoma and one for large cell neuroendocrine carcinoma. There was a broad distribution of pathological stages among these patients from IA to IIIA (Table 5). Two patients received neoadjuvant therapy prior to their index operation and six (86%) underwent anatomic lung resection. The only patient who did not receive an anatomic lung resection had a < 2 cm, stage IA large cell neuroendocrine tumor, had a therapeutic wedge resection without lymph node evaluation, and has a disease-free-survival > 15 years. No patients received adjuvant radiation therapy, whereas three received adjuvant chemotherapy. Three of the six patients who survived the operation had tumor recurrence, all requiring additional thoracic procedures. 3-year survival was 85.7% (Fig. 1). Median overall survival was 65 months.

**Table 5:** List of patients with non-small cell lung cancer undergoing therapeutic lung resection

Gender	Age	Year of operation	Type of surgery	Initial operative approach	Convert to open	Diagnosis	Neoadjuvant therapy	Lymph node sampling	Pathologic stage	Adjuvant chemotherapy	Adjuvant radiation therapy	Recurrence	Time to recurrence (months)	Reoperation	Length of survival (months)
F	70	2005	Left upper lobe wedge resection	VATS	No	Large cell neuroendocrine carcinoma	No	Not done	IA	No	No	None	N/A	None	184 (alive)
F	73	2007	Left upper lobectomy	Thoracotomy	N/A	Adenocarcinoma	Yes	5, 7, 9L	IIIA	Yes	No	None	N/A	None	143
F	65	2009	Right lower lobectomy	VATS	Yes	Adenocarcinoma	No	4R, 7, 9R, 11R	IIB	Yes	No	Distant	16	Thoracotomy, LLL wedge resection MLND	45
F	47	2009	Left lower lobectomy	VATS	Yes	Adenocarcinoma	No	5, 7, 10L, 11L	IIB	No	No	Distant	18	VATS, RUL wedge resection	87
F	74	2012	Left lower lobectomy	VATS	No	Adenocarcinoma	No	5, 6, 9L, 11L	IB	No	No		47	Left VATS, pleural biopsy, TPC placement	65
M	73	2019	Left upper lobectomy	VATS	Yes	Adenocarcinoma	Yes	5, 7, 8L, 9L, 11L, 12L	IIA	N/A	N/A	Distant	N/A	None	0
F	58	2020	Right lower segmentectomy	VATS	No	Adenocarcinoma	No	4R, 7, 9R, 10R, 11R	IA	No	No	None	N/A	None	14 (alive)

*VATS* video-assisted thoracic surgery; *N*/*A* not applicable; *LLL* left lower lobe; *MLND* mediastinal lymph node dissection; *RUL* right upper lobe; *TPC* tunneled pleural catheter

## Comment

Less than 1% of the US population is comprised of Jehovah’s Witnesses (JW) [7], a group of people that refuses blood product transfusions based on their religious beliefs. This can pose important healthcare and ethical challenges during surgery [8]. There is a paucity of reported outcomes of thoracic surgery in this population, with only isolated case reports found in the English literature. One case report and one case series have been reported from Japan. Nishomoto et al. reported a case of left lower lobectomy for pulmonary adenocarcinoma without blood transfusion in 1998, and went into deep depth to highlight their preoperative consultation [9]. Takagi et al. recently reported a series of ten JW patients with lung tumor resection at a single center in Japan and reported no serious complications, except for a single patient with anemia due to postoperative hemorrhagic gastrointestinal ulcer [8]. However, lung cancer is the leading cause of cancer-related death in the United States and throughout the world and therefore one would assume JW patients present each year for lung resections and are potentially denied resection due to uncertainty of outcome. Consequently, a need exists to document surgical outcomes of lung resection in JW patients to better understand their perioperative risk and facilitate shared decision making. Thus, the aim of this study is to review perioperative outcomes and long-term cancer survival of JW patients undergoing pulmonary resection.

A wide variety of lung resections were performed in JW patients at our center in the past 20 years, both anatomic and non-anatomic and for benign and malignant disease. Operative mortality in this series was 6%. The patients with NSCLC who underwent curative lung resections, and survived their index hospitalization, had disease free survival of at least 14 months, with the longest disease-free survival of 15 years. Peri-operative data noted a median Hgb decrease of 2.2 among JW patients who underwent anatomic lung resections, which was acceptable, especially considering that the average preoperative Hgb value of the cohort was 11.9. Of note, two patients undergoing anatomic resection in 2020 (segmentectomy and lobectomy) did not have pre- or postoperative Hgb levels available, reflecting our current practice of not routinely checking perioperative Hgb levels in general thoracic surgery patients unless there is a clinical indication that would change management.

This series identified a higher rate of conversion from VATS to thoracotomy among patients who underwent lung resection for NSCLC compared to other diagnoses, at 50% vs. 0%. The 50% conversion rate for anatomic NSCLC resections is high, considering unexpected VATS conversion to thoracotomy was reported in 2.5% to 23% of the cases in the literature [10–12]. This could be due to the combination of early avoidance of the dreaded scenario of major blood loss in JW patients and the more difficult hilar dissection in advanced stage NSCLC, as two in four of our conversion cases were due to concern of difficult dissection in JW patients with locally advanced adenocarcinoma. In our series, one in four of the conversion cases was due to major vascular injury, similar to that reported in the literature at 21% to 29% of all conversion cases [10–12], suggesting equivalent surgical skills of our surgeons. This patient underwent attempted VATS lobectomy for stage IIB left upper lobe adenocarcinoma. The incidence suggested that when operating on JW patients with more complex and advanced stage of the tumor, a lower threshold for early conversion to open approach may be prudent. Other common causes of conversion cases in the literature include hilar calcification, tumor size, tumor invasion or extension, pleural adhesion, incomplete interlobar fissure, and failure of single-lung ventilation [10–12]. In our series, half of the cases that were converted to open were converted immediately following initial video inspection of the chest. Perhaps the technical “take home message” of this study would be the appropriateness of minimally invasive approach for lung resection in JW patients as well as the awareness to convert early if there is anticipated difficult hilar dissection to avoid major blood loss.

In the single mortality in this series, cell saver was used during the procedure. Cell saver and cardiopulmonary bypass and are specifically addressed as techniques used for “bloodless surgery” by the JW Hospital Liaisons committee and in The Watchtower, the monthly religious magazine, published by the Watch Tower Bible and Tract Society that serves as an official means of disseminating JW beliefs [13]. Most members of the JW group accept auto-transfusion of blood provided aiding device for auto-transfusion (e.g. a cell saver) is attached in a closed system connected to their circulation and that the blood is not stored [14, 15]. Acceptance of these technologies is left to the conscience of the individual as to whether he or she believes that the bypass pump or the cell salvage machine tubing is truly an extension of themselves. These preferences should be addressed and carefully outlined in the surgical consent prior to the procedure. As a referral center for JW care, our hospital has a unique operative consent which specifically addresses the individual wishes with regard to blood expanders and these technologies.

Alternatives to allogenic blood may be considered and should also be addressed. There may also be a theoretical benefit of preoperative erythropoietin (EPO) treatment to increase Hgb levels prior to elective surgery. Unfortunately, the need for timely resections for a cancer diagnosis makes this strategy unattractive for many pulmonary resections and no patients in this series received EPO treatment. Studies available to date have not shown clinically meaningful differences in the outcomes between matched cohort studies of patients declining transfusion and receiving EPO matched to control patients declining transfusion and not receiving EPO [16]. Other measures include preoperative vitamins (B6, B12, folic acid) and iron administration are reported in other surgical fields [17, 18].

It is our hope that the results of this study may help address some of the ethical concerns regarding the increased risk of mortality and morbidity for JW’s patients to undergo lung resection surgery. Despite the potentially increased risk of operative mortality, JW’s should not be deprived of the necessary care for lung cancer based on the false assumption that refusing blood transfusions means that they do not want to consider surgical interventions [19]. However, all parties, including surgeons, patients, and anesthesiologists, should fully understand and agree with the increased risk of surgical complications prior to operation. Rollins et al. investigated the personal experiences of six Jehovah’s Witnesses who underwent major abdominal surgery at a single institution, and reported that all patients thought that faith helped them and their families to make the decision to opt for surgical intervention and to cope if faced with a life-threatening situation. The study further reported that JW’s being well respected, understood and informed made the decision process of JW’s to undergo surgery easier [20]. JW’s also tend to be compliant with other medical treatments and are often willing to pursue other types of interventions [19]. As such, when consulting JW’s, one may consider offering other non-surgical therapy for early-stage NSCLC such as stereotactic body radiotherapy (SBRT). A meta-analysis based on 14 cohort studies reported that the odds ratio of 5-year overall survival between surgical management and SBRT for early-stage NSCLC to be 2.43, favoring surgical management [21]. Yet, operative mortality for the NSCLC cohort in our study was 11%, which is much higher than 1% reported in the general population who received lobar resection for early-stage lung cancer [4]. Given the increased risk of operative mortality from avoidance of blood products, one may thus consider offering SBRT for early-stage NSCLC to JW’s.

In conclusion, this review suggests that lung resections for NSCLC and inflammatory etiologies can be performed safely in the setting of both primary and re-operative procedures. However, surgeons, anesthesiologists, and patients need to be aware and agree to the potentially increased risk of operative mortality in lung resection for NSCLC. Given the limited sample size of our study, analysis of a larger number of cases and patient backgrounds is needed to generalize these findings to other centers.

## Data Availability

The datasets during and/or analysed during the current study are available from the corresponding author on reasonable request.
